# Regulation of Adropin by Sitagliptin monotherapy in participants with newly diagnosed type 2 Diabetes

**DOI:** 10.1186/s12902-022-01233-x

**Published:** 2022-12-07

**Authors:** Qiu Wang, Yu An, Lin Zhang, Yuanying Zhang, Guang Wang, Jia Liu

**Affiliations:** Department of Endocrinology, Beijing Chao-yang Hospital, Capital Medical University, Beijing, 100020 China

**Keywords:** Adropin, Sitagliptin, Type 2 diabetes

## Abstract

**Background:**

Adropin is a potent metabolic regulator of insulin sensitivity and glycolipid metabolism. The present study investigated the effects of sitagliptin on adropin and metabolic parameters in participants with newly diagnosed type 2 diabetes (T2D).

**Methods:**

Thirty-five participants newly-diagnosed with T2D were prescribed sitagliptin 100 mg once daily for 17 weeks. Twenty-eight age-, sex-, and BMI-matched healthy subjects were included as the control group. Adropin and clinical parameters were assessed at baseline and after treatment.

**Results:**

At baseline, serum adropin levels were lower in T2D participants than in the healthy individuals (3.12 ± 0.73 vs. 5.90 ± 1.22 ng/ml, *P* <  0.01). Serum adropin levels were significantly higher in T2D patients after sitagliptin treatment (4.97 ± 1.01 vs. 3.12 ± 0.73 ng/ml, *P* <  0.01). The changes in serum adropin levels after sitagliptin treatment were associated with the improvements of fasting blood glucose (FBG) (β = − 0.71, *P* <  0.01), glycosylated hemoglobin (HbA1c) (β = − 0.44, *P* <  0.01) and homeostatic model assessment of β-cell function (HOMA-β) (β = 9.02, *P* <  0.01).

**Conclusions:**

Sitagliptin treatment could significantly increase serum adropin levels in participants with newly diagnosed T2D. The increase in serum adropin levels could be associated with the amelioration of glucose metabolism, which might be involved in beneficial glucose-lowering mechanisms of sitagliptin.

**Trial registration:**

Clinicaltrials.gov, NCT04495881. Retrospectively registered on 03/08/2020.

## Introduction

Adropin is a unique peptide hormone first reported in 2008. It contains 76 amino acids encoded by the *Energy Homeostasis Associated (Enho)* gene and is predominantly expressed in liver and brain [1]. Some animal studies have shown that adropin is involved in the control of glycolipid metabolism and the improvement of insulin sensitivity [1–4]. Lower serum adropin levels in human have been proved to be associated with multiple metabolic disorders such as type 2 diabetes (T2D), nonalcoholic fatty liver disease (NAFLD), polycystic ovary syndrome (PCOS) and metabolic syndrome [5–8].

T2D is a chronic metabolic disorder characterized by chronic hyperglycemia due to β-cell dysfunction and insulin resistance [9]. Sitagliptin is a potent and highly selective dipeptidyl peptidase-4 (DPP-4) inhibitor for T2D therapy and acts by inhibition of DPP-4 enzyme. Moreover, sitagliptin is the most widely used, because it was the first of DPP-4 inhibitors approved by the US Food and Drug Administration (FDA) [10], and it is effective, safe and generally well-tolerated in clinical practice [11]. DPP-4 is expressed in different tissues such as liver, brain and adipose tissue [12–14]. However, to our knowledge, no studies had focused on whether and how DPP-4 inhibitors regulate serum adropin. Therefore, we investigated the changes in serum adropin levels in participants newly-diagnosed with T2D following sitagliptin treatment.

## Materials and methods

### Study design

This was a phase 4, open-label, single-arm, interventional study to evaluate the effectiveness of sitagliptin for the treatment of newly diagnosed T2D. The study was conducted in accordance with the Declaration of Helsinki and was registered on Clinicaltrials.gov on 03/08/2020 (registration number NCT04495881). The protocol was approved by the Ethics Committee of Beijing Chao-yang Hospital affiliated with Capital Medical University (2020-#-182). All enrolled participants provided written informed consent.

### Recruitment

Thirty-eight participants with newly diagnosed T2D (T2D group) were recruited from outpatients at the Endocrinology Department of Beijing Chao-yang Hospital affiliated with Capital Medical University from January 2020 to March 2020. They met inclusion and exclusion criteria, which were similar to those used in our previous studies [15]. All participants underwent medical and laboratory screening including a 75 g oral glucose tolerance test (OGTT). Eligible participants aged 18 to 65 years were newly diagnosed with T2D within the previous 3 months according to the 2014 American Diabetes Association (ADA) diagnostic criteria and they were further selected when HbA1c ≥ 7.0% and ≤ 9.5% [16]. They did not use any hypoglycemic drugs before the enrolment. The healthy control group included twenty-eight age-, sex-, and body mass index (BMI)-matched healthy participants with normal glucose tolerance who received routine physical examination. We excluded all participants whose HbA1c levels were out of our selection range, those who had type 1 diabetes, pancreatitis, pregnancy or possible pregnancy, liver or renal function impairment, coronary heart disease, infectious disease, history of intestinal surgery, chronic hypoxic diseases, hematological disease, systemic inflammatory disease and cancer, and those who had medication use related to hypertension, hyperlipidemia, hyperglycemia and other metabolic-related disorders.

The T2D participants received sitagliptin 100 mg orally once daily for 17 weeks. Furthermore, all participants were given advice for lifestyle modifications consisting of diet and physical activity. Drug compliance, vital signs and adverse events were monitored by outpatient review or telephone follow-up. The patients who had poor glycemic control (FBG values > 10 mmol/L within four weeks) or could not accept the follow-up visit were withdrawn from the study.

### Clinical and biochemical measurements

The newly diagnosed T2D participants were followed-up for 17 weeks. The fasting blood samples were taken before and after the 17-week sitagliptin treatment. The serum samples from all participants were collected in the morning after an overnight fast and stored at − 80 °C until analysis.

Anthropometric measurements and biochemical laboratory tests were performed at baseline (pre-treatment) and after 17 weeks of sitagliptin treatment (post-treatment). Weight and height were measured to the nearest 0.1 kg and 0.1 cm in the fasting state, respectively. FBG, HbA1c, fasting insulin (FINS), total cholesterol (TC), low-density lipoprotein cholesterol (LDL-C), high-density lipoprotein cholesterol (HDL-C) and triglycerides (TG) were measured at the central chemistry laboratory of Beijing Chao-yang Hospital affiliated with Capital Medical University. The primary outcome measure was the changes in HbA1c at baseline and after 17 weeks treatment, and the secondary outcome measures included changes in FBG, FINS, homeostasis model assessment of insulin resistance (HOMA-IR), HOMA-β, TC, HDL-C, TG and LDL-C.

Serum adropin levels were measured using commercial enzyme-linked immunosorbent assay (ELISA) kits (EK-032-35, Phoenix Pharmaceuticals, Inc., USA) for quantitative detection. The sensitivity of the assay was 0.32 ng/ml and the linear range of the standard was 0.32–5.80 ng/ml. BMI was calculated as the weight in kilograms divided by the height in meters squared (kg/m^2^). HOMA-IR and HOMA-β were calculated by the following formulas: HOMA-IR = FINS (μIU/mL) × FBG (mmol/L)/22.5; HOMA-β = 20 × FINS (μIU/mL)/FBG (mmol/L) − 3.5 [17, 18].

### Statistical analysis

Data analyses were performed using the SPSS statistical software, version 23.0 (SPSS Inc., Chicago, IL, USA). The normality of distribution was tested by Shapiro–Wilk test. The data were expressed as means ± standard deviation (SD) or median (interquartile range). The Student’s *t*-test or nonparametric Mann-Whitney U-test for continuous variables was used to compare the differences between healthy control group and T2D group. Statistical differences versus baseline after 17 weeks of pharmacotherapy were tested using the paired Student’s *t*-test or nonparametric Wilcoxon test. Pearson and Spearman correlation coefficients were used to assess the correlations between serum adropin levels and other metabolic parameters at baseline as appropriate. We fit linear mixed-effects models using the STATA version 13.0 (STATA, College Station, TX), allowing for the inclusion of individual as a random effect, to examine the longitudinal relationships between serum adropin levels and insulin resistance/sensitivity indicators as well as the parameters of glucose metabolism and lipid metabolism over the study period. Mixed effects linear models can account for the associations between repeated measures owing to unobserved inter-individual heterogeneity by incorporating random effects. All statistical tests were two-tailed and *P* values < 0.05 were considered statistically significant.

## Results

### Baseline characteristics of control subjects and participants with T2D

Among thirty-eight enrolled participants with T2D, thirty-five completed the follow-up and three dropped out of this study because of poor glycemic control. No hypoglycemia occurred during the follow-up period. The baseline characteristics of the study participants were summarized in Table 1. Comparative analysis of baseline characteristics showed that there were no significant differences as regards age, sex, BMI, TC, LDL-C, TG and FINS levels between healthy control group and T2D group (all *P* > 0.05). Nevertheless, higher FBG, HbA1c and HOMA-IR as well as lower HDL-C and HOMA-β were observed in the T2D group compared with the healthy individuals (all *P* <  0.05). Serum adropin levels were significantly lower in the T2D group than in the control group (3.12 ± 0.73 vs. 5.90 ± 1.22 ng/ml, *P* <  0.01).

**Table 1:** Baseline characteristics of the study participants

	Groups		***P*** value
	Matched control (***n*** = 28)	Type 2 diabetes (***n*** = 35)	
Age (year)	50.25 ± 13.49	50.31 ± 13.43	0.985
Sex (male/female)	16/12	21/14	0.819
BMI (kg/m^2^)	24.63 ± 2.88	25.67 ± 3.10	0.178
TC (mmol/L)	4.84 ± 0.99	4.80 ± 0.92	0.880
LDL-C (mmol/L)	2.92 ± 1.01	3.04 ± 0.94	0.660
HDL-C (mmol/L)	1.32 ± 0.37	1.15 ± 0.26	0.042*
TG (mmol/L)	1.44 (0.89, 2.14)	1.80 (1.24, 2.27)	0.148
FBG (mmol/L)	5.00 ± 0.61	8.67 ± 1.47	< 0.001*
FINS (μIU/mL)	8.00 (5.10, 13.25)	10.20 (6.70, 13.10)	0.316
HbA1c (%)	5.64 ± 0.47	8.04 ± 0.74	< 0.001*
HOMA-IR	1.76 (1.10, 2.99)	4.19 (2.21, 5.56)	< 0.001*
HOMA-β	120.55 (77.38, 165.15)	41.23 (22.83, 58.26)	< 0.001*
Adropin (ng/mL)	5.90 ± 1.22	3.12 ± 0.73	< 0.001*

Abbreviations: *BMI* body mass index, *TC* total cholesterol, *LDL-C* low-density lipoprotein cholesterol, *HDL-C* high-density lipoprotein cholesterol, *TG* triglyceride, *FBG* fasting blood glucose, *FINS* fasting insulin, *HbA1c* glycosylated hemoglobin, *HOMA-IR* homeostasis model assessment of insulin resistance, *HOMA-β* homeostasis model assessment of β-cell function

Data shown as mean ± standard deviation were compared between 2 groups using Student’s t test for independent samples

Data shown as median (interquartile range) were compared between 2 groups using Mann-Whitney U-test

Data shown as n (%) were compared between two groups using the chi-square test

*P <  0.05

### Correlations between serum adropin levels and the baseline parameters

In addition, the correlations between serum adropin levels and the baseline parameters were investigated in all participants. The serum adropin levels were significantly negatively correlated with TG, FBG, HbA1c and HOMA-IR (TG: *r* = − 0.271; *P* <  0.05; FBG: *r* = − 0.750, *P* <  0.01; HbA1c: *r* = − 0.770, *P* <  0.01; HOMA-IR: *r* = − 0.441, *P* <  0.01). Serum adropin levels were significantly positively correlated with HDL-C and HOMA-β (HDL-C: *r* = 0.280, *P* <  0.05; HOMA-β: *r* = 0.596, *P* <  0.01) (Table 2).

**Table 2:** Simple correlation analyses of serum adropin levels associated with other biochemical parameters in baseline

	Adropin	
	***r***	***P*** value
Age	−0.055	0.669
BMI	−0.243	0.055
TC	0.084	0.513
LDL-C	0.079	0.539
HDL-C	0.280	0.026*
TG	−0.271	0.032*
FBG	−0.750	< 0.001*
FINS	−0.152	0.236
HbA1c	−0.770	< 0.001*
HOMA-IR	−0.441	< 0.001*
HOMA-β	0.596	< 0.001*

Abbreviations: *BMI* body mass index, *TC* total cholesterol, *LDL-C* low-density lipoprotein cholesterol, *HDL-C* high-density lipoprotein cholesterol, *TG* triglyceride, *FBG* fasting blood glucose, *FINS* fasting insulin, *HbA1c* glycosylated hemoglobin, *HOMA-IR* homeostasis model assessment of insulin resistance, *HOMA-β* homeostasis model assessment of β-cell function

*P < 0.05

### Effect of sitagliptin monotherapy on serum adropin levels and other metabolic parameters in T2D group

Changes in clinical parameters after sitagliptin treatment in the T2D group are summarized in Table 3. BMI, FBG, HbA1c and HOMA-IR after sitagliptin treatment were significantly decreased compared with the baseline (all *P* <  0.01). Moreover, HOMA-β was significantly increased after sitagliptin treatment (*P* <  0.01). But no obvious changes in lipid profiles (TC, LDL-C, HDL-C and TG) and FINS were observed (all *P* > 0.05). Importantly, the increases in serum adropin levels were observed after sitagliptin treatment compared to baseline (4.97 ± 1.01 vs. 3.12 ± 0.73 ng/ml, *P* <  0.01).

**Table 3:** Pre-treatment and post-treatment clinical characteristics of T2D participants treated with sitagliptin

	Group		Changes after sitagliptin	***P*** value
	Pre-treatment (n = 35)	Post-treatment (***n*** = 35)		
BMI (kg/m^2^)	25.67 ± 3.10	25.12 ± 3.14	− 0.55 (− 0.83, − 0.26)	< 0.001*
TC (mmol/L)	4.80 ± 0.92	4.79 ± 0.85	−0.02 (− 0.23, 0.20)	0.881
LDL-C (mmol/L)	3.04 ± 0.94	3.03 ± 0.91	−0.01 (− 0.23, 0.21)	0.938
HDL-C (mmol/L)	1.15 ± 0.26	1.20 ± 0.32	0.05 (− 0.01, 0.10)	0.088
TG (mmol/L)	1.80 (1.24, 2.27)	1.39 (1.05, 2.34)	−0.08 (− 0.42, 0.27)	0.101
FBG (mmol/L)	8.67 ± 1.47	6.68 ± 0.93	−1.98 (−2.47–1.49)	< 0.001*
FINS (μIU/mL)	10.20 (6.70, 13.10)	7.80 (5.40, 12.40)	−1.60 (−3.48, 0.28)	0.177
HbA1c (%)	8.04 ± 0.74	6.59 ± 0.53	− 1.45 (− 1.73, − 1.17)	< 0.001*
HOMA-IR	4.19 (2.21, 5.56)	2.50 (1.41, 3.49)	− 1.56 (− 2.47, − 0.65)	< 0.001*
HOMA-β	41.23 (22.83, 58.26)	53.47 (32.95, 85.31)	19.29 (9.44, 29.14)	< 0.001*
Adropin (ng/mL)	3.12 ± 0.73	4.97 ± 1.01	1.85 (1.47, 2.23)	< 0.001*

Abbreviations: *BMI* body mass index, *TC* total cholesterol, *LDL-C* low-density lipoprotein cholesterol, *HDL-C* high-density lipoprotein cholesterol, *TG* triglyceride, *FBG* fasting blood glucose, *FINS* fasting insulin, *HbA1c* glycosylated hemoglobin, *HOMA-IR* homeostasis model assessment of insulin resistance, *HOMA-β* homeostasis model assessment of β-cell function

Data shown as mean ± standard deviation were compared between pre- and post-treatment using paired Student’s t test

Data shown as median (interquartile range) were compared between pre- and post-treatment using paired Wilcoxon test

*P < 0.05

### Association between serum adropin levels and metabolic parameters after sitagliptin treatment

**Fig. 1:**
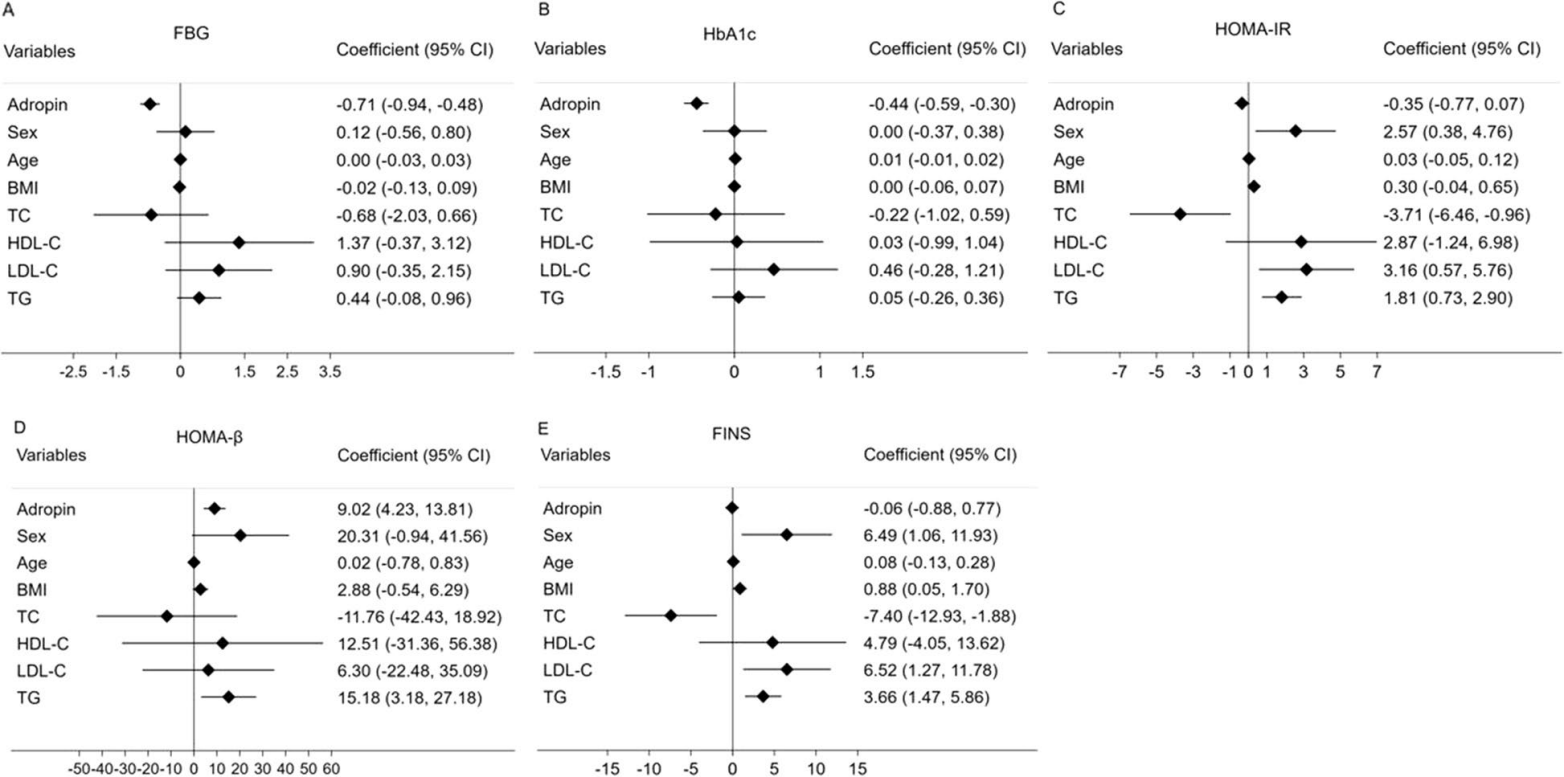
Forest plots demonstrating associations between adropin and FBG (**A**), HbA1c (**B**), HOMA-IR (**C**), HOMA-β (**D**) and FINS (**E**) in mixed-effect linear models adjusted for potential confounders. Abbreviations: FBG: fasting blood glucose; HbA1c: glycosylated hemoglobin; HOMA-IR: homeostasis model assessment of insulin resistance; HOMA-β: homeostasis model assessment of β-cell function; FINS: fasting insulin; BMI: body mass index; TC: total cholesterol; HDL-C: high-density lipoprotein cholesterol; LDL-C: low-density lipoprotein cholesterol; TG: triglyceride

**Fig. 2:**
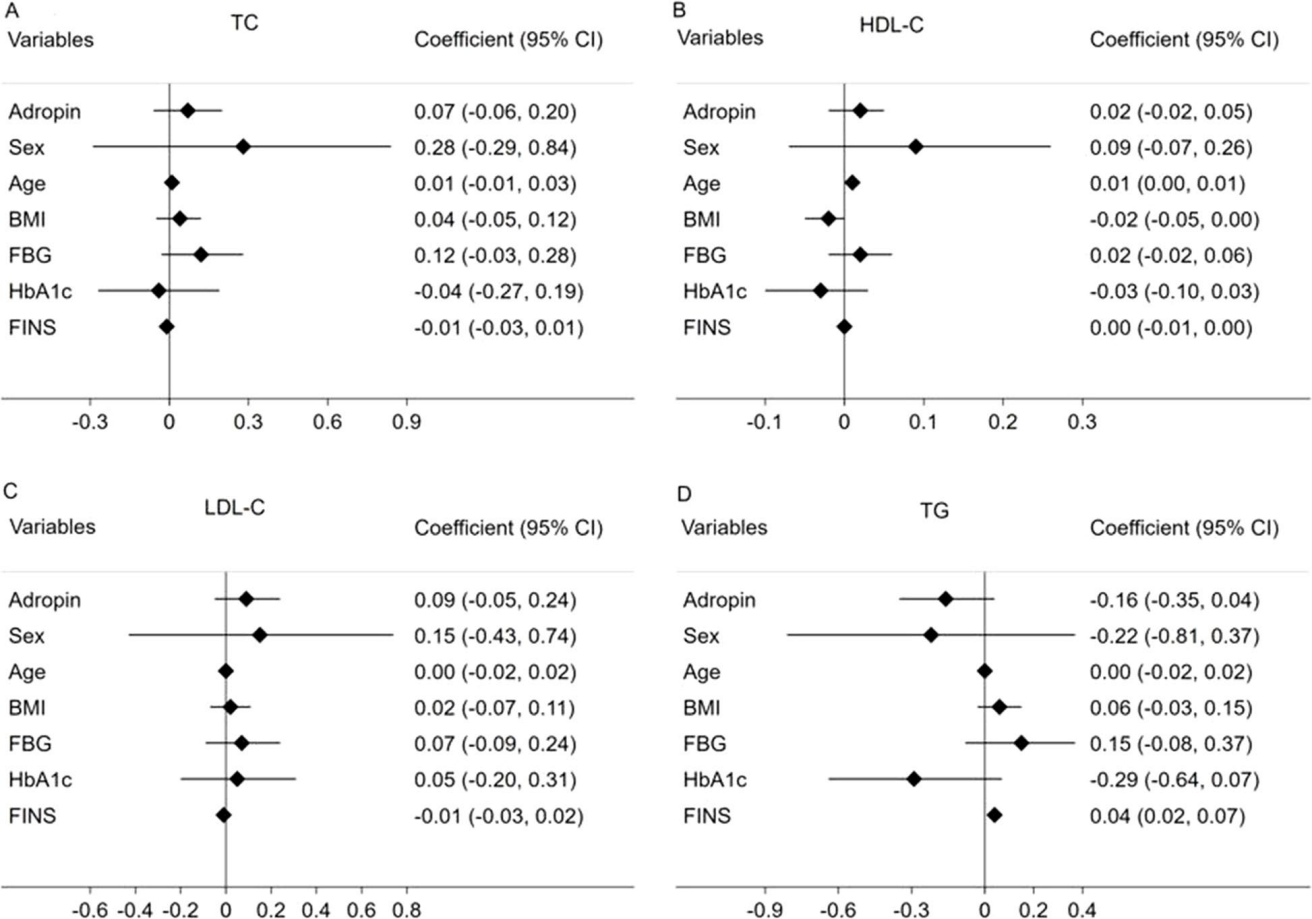
Forest plots demonstrating associations between adropin and TC (**A**), HDL-C (**B**), LDL-C (**C**) and TG (**D**) in mixed-effect linear models adjusted for potential confounders. Abbreviations: TC: total cholesterol; HDL-C: high-density lipoprotein cholesterol; LDL-C: low-density lipoprotein cholesterol; TG: triglyceride; BMI: body mass index; FBG: fasting blood glucose; HbA1c: glycosylated hemoglobin; FINS: fasting insulin

Longitudinal analyses over the study period using linear mixed-effects models were displayed in Figs. 1 and 2. In multivariable models adjusted for sex, age, BMI and lipid profiles (TC, HDL-C, LDL-C and TG), higher serum adropin levels were associated with lower FBG (β = − 0.71, *P* <  0.01, Fig. 1A), lower HbA1c (β = − 0.44, *P* <  0.01, Fig. 1B), and higher HOMA-β (β = 9.02, *P* <  0.01, Fig. 1D). The associations between HOMA-IR (β = − 0.35, *P* > 0.05, Fig. 1C), FINS (β = − 0.06, *P* > 0.05, Fig. 1E) and serum adropin levels were not statistically significant. Using other regression models adjusted for sex, age, BMI, FBG, HbA1c and FINS, lipid profiles were not significantly associated with serum adropin levels (all *P* > 0.05, Fig. 2A-D).

## Discussion

The present study showed that serum adropin levels were lower in participants with newly diagnosed T2D than in the healthy controls. At baseline, serum adropin levels were negatively correlated with TG, FBG, HbA1c and HOMA-IR while positively correlated with HDL-C and HOMA-β. Importantly, sitagliptin treatment could significantly increase serum adropin levels with the improvement of metabolic parameters. Furthermore, the associations of adropin with FBG, HbA1c and HOMA-β still existed after sitagliptin treatment.

Adropin is a regulatory hormone produced mainly in the liver and brain, which is involved in glucolipid metabolism and insulin sensitivity [2, 3]. Our study showed that the T2D participants had significantly lower adropin levels. Meanwhile, serum adropin levels were negatively correlated with TG, FBG, HbA1c and HOMA-IR while positively correlated with HDL-C and HOMA-β. These results were similar with other studies [8, 19]. In animal studies, adropin overexpression or adropin treatment significantly ameliorated insulin resistance, enhanced glucose tolerance and improved glycolipid metabolism in diet-induced obese mice with insulin resistance [3, 4, 20]. In streptozotocin (STZ)-induced diabetic rats, adropin administration could improve glycemic levels, body weight, insulin levels, hepatic glycogen and carbohydrate enzymes in a dose-dependent manner, which were almost close to those of the normal control [21]. Mechanistically, several studies have found that adropin can exert beneficial metabolic effects through different mechanisms. Firstly, it was reported that adropin had a direct role in the regulation of glucose metabolism. Adropin enhanced glucose uptake via increased translocation of peroxisome proliferator-activated receptor γ (PPARγ) and glucose transpoter-4 (GLUT-4) to the plasma membrane. Secondly, adropin exerted antidiabetic effect through the AMPK pathway, which was mainly reflected in the elevations of AMPK levels and acetyl CoA carboxylase (ACC) phosphorylation in diabetic rat liver [21]. Furthermore, adropin enhanced hepatic intracellular signaling actions that were involved in insulin-mediated glucose homeostasis. Adropin inhibited cAMP-PKA signaling pathway, leading to reduce the inositol triphosphate receptor (IP3R) phosphorylation and suppress the expression of cAMP-responsive element-binding protein (CREB) and CREB-regulated transcription co-activator 2 (CRTC2), which are two key factors in hepatic glucose metabolism [3, 4, 20]. Additionally, adropin reduced the expression of genes involved in lipogenesis such as stearoyl-CoA desaturase-1 (SCD-1) and fatty acid synthase (Fas) in both liver and adipose tissue [1]. Therefore, these findings indicated that decreased adropin was closely related to the occurrence and development of T2D.

Consistent with previous studies [22–24], FBG and HbA1c significantly decreased, and lipid metabolism was not appreciably altered in T2D participants following sitagliptin treatment. However, sitagliptin treatment exhibited a greater reduction of BMI and HbA1c, and could improve indices of insulin resistance or sensitivity in our study, which are inconsistent with several previous studies [25–28]. Several possible reasons that could explain these inconsistences. In the first place, sitagliptin was employed as the initial therapy, meanwhile, lifestyle modifications were used as an add-on treatment. As a result, the above-mentioned effects might be overlapped by the combination of both. These findings are similar to those of previous studies [29, 30]. In the second place, Chinese adults with T2D have higher postprandial plasma glucose levels compared with Western participants [31]. Sitagliptin stimulates glucose-dependent insulin secretion and inhibits postprandial glucagon effectively, thus significantly lowering the postprandial glucose levels [32]. Moreover, sitagliptin has been shown various glucose-lowering efficacies but these different effects varied from ethnic differences. A meta-analysis demonstrated that sitagliptin could increase insulin sensitivity among in Asian T2D patients than in Caucasian T2D patients [33]. These results are supported by animal studies showing that DPP-4 inhibitors can improve insulin resistance and increase insulin sensitivity [34–37].

Sitagliptin treatment significantly increased serum adropin levels, which were associated with the improvements in FBG, HbA1c and HOMA-β. In animal studies, adropin overexpression significantly reduced insulin resistance and improved glucose tolerance in obese high-fat-fed mice [1]. Besides, adropin treatment reduced blood glucose levels, HbA1c and HOMA-IR and increased HOMA-β in a rat model of T2D [20]. However, the exact mechanism by which sitagliptin increases serum adropin levels remains unclear. We found that sitagliptin and adropin had similar effects on the regulation of glucose metabolism in previous studies. DPP-4 inhibitor could suppress gluconeogenic gene expression through the inhibition of CREB phosphorylation and CRTC2 expression in mice with diabetes [4, 37]. Based on these previous findings, we believe that the upregulation of adropin might be a potential novel mechanism for beneficial effects of sitagliptin in T2D.

But several limitations in the present study should be noted. First, our study was not a randomized controlled trial, the causality between adropin and sitagliptin cannot infer. Well-designed randomized controlled trials are needed to further confirm the beneficial effects we reported. Second, our findings are limited by a relatively small sample size, so we need to expand the sample size to support. Lastly, more animal and cell experiments are needed to reveal the underlying molecular mechanism of sitagliptin on adropin.

## Conclusion

In conclusion, our study demonstrated that serum adropin levels were lower in newly diagnosed T2D participants than in healthy controls. Sitagliptin treatment could significantly increase serum adropin levels in participants with newly diagnosed T2D. The increase in serum adropin levels could be associated with the amelioration of glucose metabolism, which might be involved in underlying anti-diabetic mechanism of sitagliptin.

## Data Availability

The datasets used and/or analysed during the current study are available from the corresponding author on reasonable request.

## Abbreviations

T2D: Type 2 diabetes
FBG: fasting blood glucose
HbA1c: glycosylated hemoglobin
HOMA-β: homeostasis model assessment of β-cell function
Enho: Energy Homeostasis Associated
NAFLD: nonalcoholic fatty liver disease
PCOS: polycystic ovary syndrome
DPP-4: dipeptidyl peptidase-4
FDA: Food and Drug Administration
OGTT: oral glucose tolerance test
ADA: American Diabetes Association
BMI: body mass index
FINS: fasting insulin
TC: total cholesterol
LDL-C: low-density lipoprotein cholesterol
HDL-C: high-density lipoprotein cholesterol
TG: triglyceride
HOMA-IR: homeostasis model assessment of insulin resistance
ELISA: enzyme-linked immunosorbent assay
SD: standard deviation
STZ: streptozotocin
PPARγ: peroxisome proliferator-activated receptor γ
GLUT-4: glucose transpoter-4
ACC: acetyl CoA carboxylase
IP3R: inositol triphosphate receptor
CREB: cAMP-responsive element-binding protein
CRTC 2: CREB-regulated transcription co-activator 2
SCD-1: stearoyl-CoA desaturase-1
Fas: fatty acid synthase
GLP-1 RA: GLP-1 receptor agonist
